# Is Football or Badminton Associated With More Positive Affect? The Links Between Affects and Sports Club Membership Among French Adolescents

**DOI:** 10.3389/fpsyg.2021.735189

**Published:** 2021-12-17

**Authors:** Alexis Barbry, Annie Carton, Jérémy Coquart, Hervé Ovigneur, Camille Amoura, Williams Nuytens, Gabor Orosz

**Affiliations:** ^1^Université de Rouen-Normandie, Centre des Transformations des Activités Physiques et Sportives, Rouen, France; ^2^L’Institut des Rencontres de la Forme, Wattignies, France; ^3^Univ. Lille, Univ. Artois, Univ. Littoral Côte d’Opale, ULR 7369 - URePSSS - Unité de Recherche Pluridisciplinaire Sport Santé Société, Lille, France; ^4^Univ. Artois, Univ. Lille, Univ. Littoral Côte d’Opale, ULR 7369 - URePSSS - Unité de Recherche Pluridisciplinaire Sport Santé Société, Liévin, France

**Keywords:** adolescents, negative affect, positive, sports club membership, affective benefits

## Abstract

Prior studies extensively examined the way sports club membership can lead to beneficial affective outcomes. Prior experiments also found that team sports, intensive sports, and sports that are frequently pursued can lead to even more affective benefits. However, no prior studies examined the differences between the affective benefits of specific sports. Based on prior results, we supposed that certain sports that meet all the previously set criteria—will provide the greatest affective benefits. The present large-scale investigation examined the data of adolescents (*N* = 12,849, female = 5,812, aged between 10 and 18, *M*_age_ = 12.56 years, and *SD*_age_ = 2.00) and aimed to fill this gap. Firstly, the results showed that—although differences in affect can be found between the lack of club membership and most of the sports club memberships—the differences between the specific sports are less striking. Secondly, the sports that are associated with the highest level of positive and the lowest level of negative affectivity are not necessarily the ones expected. Finally, adolescents who practice athletics, reported the lowest means of negative, and the highest means of positive affect. However, it did not differ significantly from the results regarding the most practiced sport in France: soccer. Our results suggest that soccer as the most practice sport among French adolescents was associated with more positive affects than the majority of the 10 most licensed members French sports practiced by teens between 2008 and 2019. All in all, being a member of a sports club is associated with affective benefits, and some specific sports clubs can have some extra benefits.

## Introduction

As a parent or as an adolescent, the question might arise as: what specific sports club membership might be related to the most positive affect? Surprisingly, we have had limited information to give clear answers to this question. The present work uses a large French database and aims to provide some tips to answer them.

The positive effect of physical activity on the mental health of adolescents is unquestionable (Biddle and Asare, 2011; Donnelly et al., 2016). For example, a recent meta-analysis (Donnelly et al., 2016) found that physical activity has a positive impact on children’s cognitive functioning and it also has a positive effect on the development of some areas of the brain, such as the hippocampus (Gomez-Pinilla and Hillman, 2013), the prefrontal cortices (Kopp, 2012), or the even more specific regions of the basal ganglia (Chaddock et al., 2010, 2012) which can boost complex cognitive processes. Moreover, physical activity can not only have an immediate positive effect on the brain; if it is practiced in adolescence, it can have long-lasting positive consequences. For example, it can serve as a predictor for the level of physical activity later in adulthood (Telama et al., 2005), a metric associated with better wellbeing (Abdin et al., 2018). All in all, promoting physical activity in adolescence can have cognitive benefits and can boost practicing health-enhancing behaviors in adulthood.

In the present paper, the term affective benefit is based on Bratman et al.’s (2021) work and refers to (a) more positive affect and (b) a less negative affect as the consequence of belonging to a sports club. Besides the long-lasting cognitive benefits, meta-analyses robustly demonstrate the affective benefits of physical activities among adults and adolescents (Janssen and Leblanc, 2010; Biddle and Asare, 2011; Wiese et al., 2017; Rodriguez-Ayllon et al., 2019; Marquez et al., 2020; Bourke et al., 2021). These recent works show that engaging in physical activities or doing sports can lead to elevated positive affectivity, such as positive subjective experiences or positive mood, e.g., joy, interest, and alertness (Miller, 2011). It can also reduce negative affectivity (Zhang et al., 2020).

### Characteristics of Physical Activities and Affective Benefits

Both physical and *sports activities* (referring to physical effort guided by specific rules, very often pursued competitively) appear to be associated with physical and mental health benefits. These benefits include affective consequences, better wellbeing, and better quality of life compared to other forms of leisure-time physical activities in adolescence (Eime et al., 2013). Moreover, participation in *organized sports* can have further benefits as it supports social belonging and bonding between members (Eime et al., 2013). For example, Brettschneider (2001) found that participating in organized sport (as a member of a sports club) did not only have a positive effect on adolescents’ self-esteem but it also contributed to the development of their social competence (Howie et al., 2010). Sports activities are not an encapsulated in all adolescents’ life. Gisladottir et al. (2013) found that those adolescents who were members of a sports club had stronger beliefs in performing well at school compared to their peers who did not practice sports in a club.

Organized sports have various subcategories, and prior scientific investigations focused on their different affective benefits. Pluhar et al. (2019) found that people practicing *team sports* (soccer, football, and hockey) reported less anxiety and depression compared to those who practiced *individual sports* (running, gymnastics, and diving). Robust results (Guddal et al., 2019) suggest that the mental health benefits of team sports are related to the social aspects, such as social belonging and being part of a team (e.g., Eime et al., 2013).

Another categorization of organized sports is related to the *esthetic characteristics* of the different sports. Davison et al. (2002) compared the esthetic sports (swimming, dance, gymnastics, aerobics, cheerleading, baton twirling, and figure skating) vs. the non-esthetic sports (team sport, athletics, tennis, and martial arts) and found that practicers of esthetic sports are more at risk to have concerns about their weight, which is associated with negative emotions (Augestad and Flanders, 2002; Espeset et al., 2012; Tan et al., 2016).

A third classification of organized sports is related to the *place of practice* (Thompson Coon et al., 2011). Solid evidence suggests that practicing physical activity in an outdoor environment was associated with a decrease in anger, anxiety, and depression and with an increase of energy, revitalization, and positive engagement (Thompson Coon et al., 2011; Lawton et al., 2017; Pasanen et al., 2018). Thus, team and non-esthetic sports and also practicing physical activity in a natural environment seem to be associated with less negative and more positive ones. However, the information about the impact of a specific sport on negative and positive affects throughout adolescence is lacking.

A fourth classification is related to the *intensity* of organized sports that can impact both negative and positive affectivity (Costigan et al., 2019; Howie et al., 2020).^1^ In their definition of physical activity, the World Health Organization defines three levels of intensity activities can be pursued at: low, moderate, and vigorous. Earlier studies showed that all intensity levels had certain affective benefits (Ekkekakis et al., 2000; Ekkekakis, 2003). However, more recent studies (Costigan et al., 2019; Howie et al., 2020; Qin et al., 2020) suggested that vigorous physical activity is associated with more robust psychological benefits compared to low physical activity. For example, Howie et al. (2020) found that participating at moderate or vigorous physical activities was associated with a more favorable mental health profile. Costigan et al. (2019) found that only vigorous physical activity was associated with wellbeing among adolescents. Besides intensity, two-related aspects can also have positive consequences: higher *frequency of practice* (Hassmén et al., 2000; Jiang et al., 2021) and the higher *volume of physical activity* (Sagatun et al., 2007; Bell et al., 2019) both lead to better affective benefits.^2^

### The Present Study

One can suppose that if adolescents can harvest the affective benefits of doing sports, they will be motivated to engage more in sports to enjoy the life-long positive consequences. Therefore, it is important to know what type of sports might be associated with the strongest positive affects and least frequently with negative ones. Based on the above-mentioned studies, we expected that sports club membership in general will have a robust affective benefit compared to the lack of membership. However, we also expected that membership of a club for a collective, non-esthetic, outdoor, and vigorous sport will lead to the most salient affective benefits. It seems unlikely that a single sport will fulfill all of these criteria. However, some sports could meet most of them, e.g., soccer, athletics, and basketball. In contrast, gymnastics, dance, or horse riding meet the least of these criteria.

## Materials and Methods

### Procedure and Participants

This study was conducted in accordance with the Declaration of Helsinki and with the approval of the National Ethics Committee Board (n°00012476-2021-28-05-109). Participants for this study were recruited through the project of the so-called “*French Physical and Mental Health Inventory*” program. Besides the National Ethics Committee permission, the data gathering, and its further use was approved by the National Commission on Informatics and Liberty (RF 1232206). As the respondents were minors, written and signed informed consents were obtained from their parents. Potential respondents of the survey were informed about the content of the research and they were requested to indicate their intention to participate (they provided an assent).

The dataset was part of an extensive multi-year, cross-sectional data gathering. For the present paper, we only focus on the data of adolescents between 10 and 18 years of age. Adolescents needed to state whether they are members of a sports club or not. We have selected the adolescents who are not involved in a sports club and those who are members of a club for the most practiced sport (i.e., at least 240 adolescents per sport). We found this step important for avoiding extreme means as the result of a few biased respondents. Although the present sample is comprehensive, it was not representative for French adolescents. It was recruited between 2008 and 2019, consisted of 12,849 adolescents (5,812 females, 45.23%) who were aged between 10 and 18 years (*M* = 12.56, *SD* = 2.00). Most of them, 78.53% reported that they belong to a sports club, and approximately one fifth of the participants did not belong to a one.

### Measures

#### Positive and Negative Affects

The survey was administered approximately 30 min after a physical fitness evaluation in school, in a classroom environment. Among other measures, participants were requested to describe the extent they experienced a set of positive and negative emotions in the past three or four days. Seven adjectives described negative affects (angry, sad, anxious, ashamed, guilty, annoyed, and worried), and five described positive ones (joyful, enthusiastic, proud, full-of-energy, and happy). Responses were provided on a five-point Likert-scale ranging from 1 (not at all) to 5 (very much). This questionnaire has been recently used by Carton et al. (2021). We supposed that the 12 items belong to two factors. A confirmatory factor analysis (performed with R, package lavaan) was conducted with two first-ordered factors, with robust maximum likelihood estimator (MLR) supported this notion with acceptable model fit indices (CFI = 0.941, TLI = 0.927, RMSEA = 0.064, CI_95%_ = 0.062–0.066, SRMR = 0.039). Although these values are not perfect, they are acceptable based on the seminal work of Hu and Bentler (1999) and Marsh et al. (2005). Both the positive (*α* = 0.85) and negative scales (*α* = 0.79) have excellent internal consistency values.

#### Sports Club Membership

Before the measurements of physical fitness level and after the measurements of height and body mass, the participants responded to the following question: “Are you a member of a sports club?.” The participants who answered positively to this question were asked to report the sport they practiced most regularly.

## Results

### Analytic Strategy

The normality of the distribution was verified with a Shapiro–Wilk test, and equality of variances was analyzed with Levene’s test. One-way ANOVA with Benjamini-Hochberg post-hoc test was conducted to determine differences in positive and negative affects related to the different sports. This method uses a modified version of the Bonferroni correction for a high number of hypothesis testing. As in the present case controlling for the false discovery rate was more important than the conservative control of familywise error rate, this post-hoc approach appeared adequate (Benjamini and Hochberg, 1995). Based on the recommendations and prior studies, this post-hoc method is strongly recommended if the number of tests is high and it is broadly used in numerous recent studies that conducted similar, large-scale multiple comparisons (Nakajima et al., 2019; Hopkins et al., 2020; Maroun et al., 2021).

### Negative Affect Differences Along Sport Club Membership

**Figure 1:**
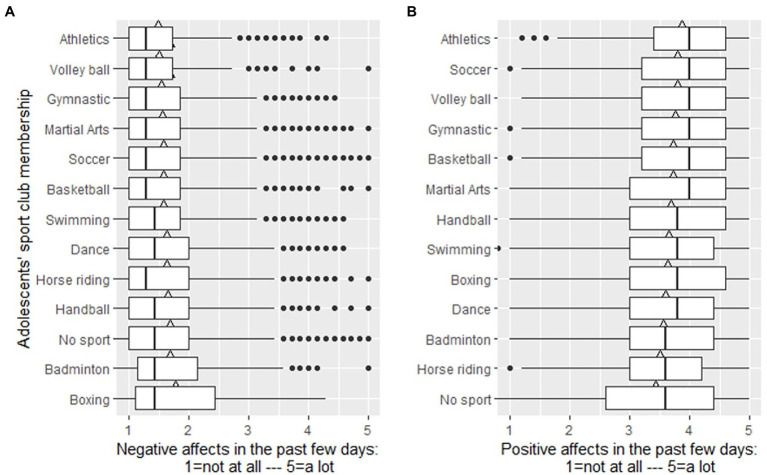
Negative and positive affects along the most practice French sports club memberships and those who do not practice sports among adolescents aged between 10 and 18 years. The left panel **(A)** depicts negative affect-related results. The right panel **(B)** depicts the main effect of the sports club membership on positive affects. Only those sports are depicted that were mentioned by at least 240 adolescents. The line within the boxes indicates the median value, while the triangle on the top of the boxes indicates group means, and the dots indicate extreme values (outliers).

ANOVA post-hoc analyses demonstrated differences between 44.9% of the comparisons of the sport club memberships that French teens frequently mentioned. Teens practicing martials arts, athletics, basketball, soccer, gymnastics, swimming, and volleyball reported significantly lower levels of negative affects compared to their peers without a sports club membership. We would like to highlight only a few examples of the inter-sport differences. For example, teens who practice boxing reported more negative affect compared to those who practice athletics, volleyball, gymnastics, martial arts, soccer, basket, swimming, dance, horse riding, and handball. Badminton club members and those teens who do not belong to any sports club reported more negative affect compared to those who practice athletics, martial arts, basketball, soccer, gymnastics, and volleyball. However, teens who practice athletics, gymnastics, soccer, and volleyball reported less negative affect compared to those who practice badminton, boxing, handball, or to those who did not practice in a sports club. See Table 1 and Figure 1 for details about differences in negative affects.

**Table 1:** Post-Hoc differences concerning negative affects along each sport.

	Athletics	Badminton	Basketball	Boxing	Dance	Gymnastics	Handball	Horse Riding	Martial Arts	Soccer	Swimming	Volleyball
Badminton	0.002^*^	–	–	–	–	–	–	–	–	–	–	–
Basketball	0.113	0.049^*^	–	–	–	–	–	–	–	–	–	–
Boxing	<0.001^*^	0.228	<0.001^*^	–	–	–	–	–	–	–	–	–
Dance	0.005^*^	0.318	0.151	0.010^*^	–	–	–	–	–	–	–	–
Gymnastics	0.384	0.016^*^	0.518	<0.001^*^	0.037^*^	–	–	–	–	–	–	–
Handball	0.003^*^	0.599	0.080	0.049^*^	0.599	0.022^*^	–	–	–	–	–	–
Horse riding	0.010^*^	0.386	0.211	0.021^*^	0.970	0.063	0.069	–	–	–	–	–
Martials Arts	0.262	0.018^*^	0.599	<0.001^*^	0.395	0.870	0.023^*^	0.073	–	–	–	–
Soccer	0.105	0.018^*^	0.823	<0.001^*^	0.026^*^	0.568	0.021^*^	0.076	0.682	–	–	–
Swimming	0.122	0.054	0.992	<0.001^*^	0.180	0.518	0.096	0.221	0.599	0.823	–	–
Volleyball	0.992	0.010^*^	0.216	<0.001^*^	0.025^*^	0.493	0.016^*^	0.034^*^	0.385	0.221	0.220	–
Non-Sport club	<0.001^*^	0.926	0.002^*^	0.078	0.100	<0.001^*^	0.518	0.223	<0.001^*^	<0.001^*^	0.004^*^	0.001^*^

*The table depicts the values of p. We considered significant differences between sports in the case of p < 0.05*.

* *Significantly different.*

### Positive Affect Differences Along Sport Club Membership

ANOVA post-hoc analyses demonstrated differences between 47,4% of the comparisons of the sports club memberships French teens frequently mentioned. Teens without a sports club membership reported significantly lower levels of positive affects compared to teens with the following ten sports club memberships: martial arts, athletics, basketball, boxing, dance, soccer, gymnastics, handball, swimming, and volleyball. We would like to highlight only a few examples of the inter-sport differences. For example, teens who practice athletics reported more positive affect compared to those who practice badminton, basketball, boxing, dance, horse riding, handball, swimming, or to those teens who do not belong to any sports club. Soccer club members reported more positive affect compared to those who practice badminton, boxing, dance, horse riding, handball, swimming, and to those who do not belong to any sports club. However, teens who practice badminton, dance, horse riding, and those who do not belong to any sports club reported less positive affect compared to those who practice athletics, soccer, volleyball, and martial arts. See Table 2 and Figure 1 for details of differences in positive affects.

**Table 2:** Post-Hoc differences concerning positive affect according to each sport.

	Athletics	Badminton	Basketball	Boxing	Dance	Gymnastics	Handball	Horse Riding	Martial Arts	Soccer	Swimming	Volleyball
Badminton	<0.001^*^	–	–	–	–	–	–	–	–	–	–	–
Basketball	0.049	0.040^*^	–	–	–	–	–	–	–	–	–	–
Boxing	0.006^*^	0.474	0.238	–	–	–	–	–	–	–	–	–
Dance	<0.001^*^	0.704	0.013^*^	0.593	–	–	–	–	–	–	–	–
Gymnastic	0.126	0.0247	0.710	0.154	0.008^*^	–	–	–	–	–	–	–
Handball	0.010^*^	0.192	0.427	0.606	0.173	0.286	–	–	–	–	–	–
Horse riding	<0.001^*^	0.403	<0.001^*^	0.087	0.100	<0.001^*^	0.007^*^	–	–	–	–	–
Martial Arts	0.039^*^	0.049^*^	0.900	0.266	0.018^*^	0.655	0.486	<0.001^*^	–	–	–	–
Soccer	0.286	<0.001^*^	0.126	0.012^*^	<0.001^*^	0.341	0.016^*^	<0.001^*^	0.093	–	–	–
Swimming	0.002^*^	0.313	0.219	0.837	0.3412	0.137	0.690	0.018^*^	0.259	0.002^*^	–	–
Volleyball	0.403	0.033^*^	0.517	0.141	0.025^*^	0.700	0.251	0.001^*^	0.474	0.837	0.141	–
Non-Sport club	<0.001^*^	0.061	<0.001^*^	0.002^*^	<0.001^*^	<0.001^*^	<0.001^*^	0.259	<0.001^*^	<0.001^*^	<0.001^*^	<0.001^*^

*The table depicts the values of p. We considered significant differences between sports in the case of p < 0.05*.

* *Significantly different*.

## Discussion

What specific sport might be associated with the highest level of positive and the lowest level of negative affects? First of all, in line with robust prior findings, most of the sports appear to have affective benefits. Secondly, the differences between these benefits are not huge and they are mostly consistent with prior findings that focused on sport characteristics as team vs. individual, esthetic vs. non-esthetic, indoors vs. outdoors, and low vs. high intensity. However, there are some nuances, limitations, and questions that might be worth discussing.

### Sports Club Participation

In a recent article, Carton et al. (2021) found that adolescents who are members of sports clubs reported more positive affects and less negative ones compared to their peers who do not belong to a sports club. The utilized affect measure is described in details in Carton et al. (2021). Practicing sports has two direct effects on the individual’s mood: (a) a general improvement in the mood right after a training session and (b) a decrease in negative emotional states, such as anxiety, irritability, and guilt (Iacolino et al., 2017). However, there are potential indirect effects, as well. For example, Putnam (2000) used a sociological perspective to point out the reasons why bowling alone is much less beneficial than doing it in the company of acquaintances. Being part of sports clubs can provide not only great opportunities to build weak links (i.e., less intimate and less in-depth relationships with the opportunity of networking), but it can also build strong links as the result of years-long friendships strengthened under stressful and exciting situations (e.g., Granovetter, 1973). These clubs can provide opportunities for adolescents to build, extend, and reinforce their social network (Howie et al., 2010); satisfy their need to belong (Baumeister and Leary, 1995; Walton and Brady, 2020), raise their self-esteem, and strengthen their social skills (Brettschneider, 2001). All of these factors can play a role in the well-known fact that adolescents who belong to a sports club experience more positive and less negative emotions (Brettschneider, 2001; Howie et al., 2010; Gisladottir et al., 2013). One might think that it does not matter what kind of sports club a teen belongs to. The present study demonstrated that it is not the case, and there are some small, but measurable differences between the benefits of pursuing different sports.

### Sports With More Affective Benefits Than Others: Athletics

All in all, the differences between the benefits of different sports were not large (never reaching *d* = 0.5), but some of them appeared to be more beneficial than others. In the following, we will examine one sport in detail that appears to have one of the greatest affective benefits: athletics. It is often considered as one of the oldest sports, tracking back to ancient Greece (776 BC). At that time, athletes were considered idols who master the harmony of body and mind (Jaeger, 1988). Adolescents belonging to an athletics club reported the highest positive and lowest negative affects among the examined groups. Although, the mean affects did not differ from other sports significantly. Athletics (also called Track and Field) are a comprehensive collection of specific sports offering a repertoire of different practices (e.g., races, jumps, and throws). The current affective results are somewhat surprising as it is an individual sport, and prior studies suggest that team sports lead to more psychological benefits compared to individual sports (Guddal et al., 2019; Pluhar et al., 2019). Despite athletic sports being individual *per se*, the value of collectivity and the team is especially important at a club level.

In line with the literature, athletics are practiced outdoors and have the extra affective benefits compared to indoors sports (Thompson Coon et al., 2011; Lawton et al., 2017; Pasanen et al., 2018). They are also often considered sports requiring vigorous physical activity that has an additional affective benefit (Costigan et al., 2019; Howie et al., 2020; Qin et al., 2020). Teens in athletics clubs participate in competitions which reflect a high level of motivation (Gernigon, 1998), contributing to positive affects (Pekrun et al., 2002). To perform well in athletics, frequent and regular practice (at least three training sessions per week) are required, also adding a tremendous amount of wellbeing benefits (Hassmén et al., 2000). Athletics appear to accumulate multiple sources of affective benefits, such as the possibility to autonomously choose achievement goals; regular, frequent, and intense practice; and an outdoor environment. We can hardly find this constellation with other types of sports.

### Sports With More Affective Benefits Than Others: Soccer

Our results also suggested that adolescents who practice soccer also experience greater affective benefits compared to other sports. This result is in line with prior studies that found team sports lead to extra affective benefits compared to individual sports (Guddal et al., 2019; Pluhar et al., 2019). Moreover, soccer is very often considered as a vigorous physical activity with a frequent aerobic intermittent exercises (Fernandes et al., 2015). Soccer is very practice among teen boys in France (Ministère de la Jeunesse des Sports et de la Vie associative, Institut national du sport et de l’éducation physique, et al., 2018). Furthermore, during adolescence, boys might have more positive affects and less negative ones compared to girls (Pascual et al., 2012). The popularity of soccer is unquestionable in France with its 2 million soccer club players today. There are one million matches played each year and tens of thousands of clubs (Ministère de la ville de la jeunesse et des sports, 2015). For these millions of players, playing soccer can provide a great opportunity to experience both positive and negative emotions, gather collective and individual experiences, and be part of just and unjust processes that can serve as a basis of recognition and disregard (Nuytens, 2011). The soccer club can strengthen the sense of social belonging in the local community and can provide various social resources (Retière, 2003). Finally, we might acknowledge that soccer can also provide room for strong identification with the famous French national team and with the very popular local teams. Soccer is also an outdoor sport which is a predictor of beneficial affective outcomes (Thompson Coon et al., 2011; Lawton et al., 2017; Pasanen et al., 2018).

To a certain extent, the present results might also contribute to the explanation of why soccer is the most popular sport in France (Ministère de la Jeunesse des Sports et de la Vie associative, Institut national du sport et de l’éducation physique, et al., 2018). Soccer could be considered a mass sport (Ohl, 2004), and based on the present results, being a member of a soccer club is associated with positive affects. All in all, it appears that for an adolescent being part of a soccer club in France does not only provide some odds to reach the highest performance in this sport (France is very often among the best teams in the world), but it could also be related to a great deal of positive affective benefits.

Our results concerning the sports with more affective benefits (i.e., athletics and soccer) can be explained by including various factors from several theoretical fields with particular emphasis on psycho-social aspects, developmental, and physiological approaches but more studies are needed to confirm and fully explain our results.

### Sports With Less Affective Benefits

Every sports club membership is associated with some level of affective benefits, as previously established. However, according to the present results, some sports seem to present somewhat less affective benefits. Our results suggest that adolescents, who practice badminton, boxing, horse riding, or dance as members of a sports club reported the least positive and the most negative affects. Prior studies can provide some hints why these sports might be associated with less affective benefits. Badminton, boxing, and dance are indoor sports which might lead to less affective benefits (Lawton et al., 2017). Badminton is a zero sum, open-skill sports in which players need to adapt their actions to quickly changing and relatively unpredictable conditions (Di Russo et al., 2010). Boxing requires inter-individual confrontation (Ministère de l’Education Nationale et de la Jeunesse, 2019) involving punches that lead to not only physical pain, but also negative emotions, such as anger or fear (Lecroisey, 2017). Boxers often experience intense negative mood the results of harsh dietary strategies to lose weight before competition (Lane and Terry, 1996; Hall and Lane, 2001). Martial arts are different from boxing as they are rooted in the local culture roots and most of them put emphasis on serenity and calm (Fournier, 2000). Dance as an esthetic sport might be linked with an increase level of weight concern (Davison et al., 2002), and this weight concern might be associated with the more negative emotions (Augestad and Flanders, 2002; Espeset et al., 2012; Tan et al., 2016). Furthermore, three-quarter of the adolescent members of dance clubs are girls, who tend to report more negative emotions and less positive ones compared to their boy peers (Ministère des Sports, 2002; Pascual et al., 2012). Although these guesses are based on prior literature, further research is required to explain these sports club-related differences among adolescents.

### Limitations

This study has some limitations. First, regarding every single student, affects were surveyed 30 min after a physical fitness assessment that could possibly—although homogenously—influence the retrieval of respondents’ affects regarding the previous few days. Adolescents could only indicate one sports club membership. If they belonged to multiple clubs simultaneously, they were requested to indicate the one they spent more time practicing with. Next time, we might provide room to indicate multiple memberships to have a deeper understanding of the cumulative effects of sports club membership. Furthermore, as the goal was keeping the survey as short as possible, we did not have an opportunity to assess the perceived intensity, frequency, and volume of the practiced sport. Although our affect measure showed consistent factor structure and great internal consistency. Other, more broadly validated tools could have been used in the current study, such as the PANAS (Watson et al., 1988). Future work might either use a more commonly utilized measure or it is also possible to validate this measure. It is also possible that future studies might try to use ecological momentary assessment (Trull and Ebner-Priemer, 2013) if they are interested in short term and less pervasive benefits. As it was a cross-sectional survey study, we do not know anything about causality. Therefore, it is entirely possible that the findings are due to self-selective bias namely, that teens with a more positive disposition tend to choose athletics or soccer and that it is not necessarily athletics or soccer that will lead to an increase in positive affects and a decrease in negative affects. Causality can only be investigated through longitudinal studies and future experimental work. Despite we aimed to choose only those sports that were practiced by many adolescents, we can mention another limit of this study regarding the unequal number of adolescents in the different groups.

## Conclusion

Our study suggests that being a member of a sports club is associated with affective benefits for teenagers. However, it appears that athletics and soccer club membership might be associated with the highest levels of affective benefits, whereas boxing, badminton, dance, or horse riding club memberships are associated with less benefits. The present one is a pioneering, large-scale study that might provide broader guidelines for future scientific investigations in this field. However, it is without establishing a unidirectional causal link stating that certain sports club membership lead to more affective benefits than others.

## Data Availability Statement

The datasets presented in this article are not readily available because the data is the intellectual property of a private association (Institut des Rencontres de la Forme, IRFO) and permission has to be requested from the IRFO before access can be granted. Requests to access the datasets should be directed to Alexis Barbry (a.barbry@irfo.fr) and should indicate the reason for which you wish to use them.

## Ethics Statement

The studies involving human participants were reviewed and approved by Institutional Review Board of 00012476-2021-28-05-109. Written informed consent to participate in this study was provided by the participants’ legal guardian/next of kin. Informed consent was obtained from all participants included in the study.

## Author Contributions

CA, AB, AC, JC, HO, WN, and GO contributed to the study design, literature review, data gathering, manuscript writing, and to the data analyses and interpretation. CA, AB, AC, JC, WN, and GO contributed to the manuscript writing. All authors commented on the draft and contributed to the final version, approved the publication of the manuscript, and agreed to be accountable for all aspects of the work.

## Funding

GO was supported by the Young Researcher STARS grant from Conseil Régional Hauts de France. AB was supported by the CIFRE n°2020/0331 grant from “Association Nationale Recherche Technologie” (ANRT).

## Conflict of Interest

The authors declare that the research was conducted in the absence of any commercial or financial relationships that could be construed as a potential conflict of interest.

## Publisher’s Note

All claims expressed in this article are solely those of the authors and do not necessarily represent those of their affiliated organizations, or those of the publisher, the editors and the reviewers. Any product that may be evaluated in this article, or claim that may be made by its manufacturer, is not guaranteed or endorsed by the publisher.

## Footnotes

^1^We have to mention that it is not only related to organized sports, but also any sort of physical activities.

^2^Hassmén et al. (2000) found that practicing regular physical activity (two to three times a week) decreased negative affect (depression, anger, and stress) but also increased wellbeing compared to practicing less frequently. Moreover, in a recent study, Jiang et al. (2021) found that children and adolescents who practiced training sessions three to five times per week have a better mental health compared to those who practice only once a week. A strongly-related element to take into account to impact the adolescents’ affect is the *volume of physical activity* (Sagatun et al., 2007; Bell et al., 2019). Indeed, increasing the volume of physical activity might be a key element of protection against emotional problems during adolescence (Bell et al., 2019).
